# A qualitative study exploring the role of perfectionism in trichotillomania

**DOI:** 10.1111/papt.12597

**Published:** 2025-05-16

**Authors:** Amy L. Zarandi, Josie F. A. Millar, Erin Waites, Judith L. Stevenson

**Affiliations:** ^1^ Department of Psychology 10 West, University of Bath Bath UK; ^2^ School of Psychology & Neuroscience University of Glasgow Glasgow UK

**Keywords:** hairpulling, perfectionism, qualitative, reflexive thematic analysis, trichotillomania

## Abstract

**Objective:**

Trichotillomania (TTM) is a condition in which individuals repeatedly pull out their hair despite adverse consequences. Several models have suggested underlying processes, with a central feature of hairpulling being an external means to regulate internal states. The frustrated action model suggests that certain affective states arise from perfectionistic beliefs and an overactive, overprepared style of planning, triggering episodes of hairpulling. To date, there is limited research investigating perfectionism in people with TTM. The current study investigated the experiences of hairpulling behaviour in people with TTM, with specific attention given to perfectionism.

**Design:**

A qualitative methodology was employed.

**Method:**

Twenty participants completed online screening questionnaires and were interviewed via an online communication platform. Data were analysed using reflexive thematic analysis.

**Results:**

The findings identified the role and development of maladaptive perfectionism in hairpulling. Furthermore, a maintenance cycle is proposed, in which hairpulling is maintained via the positive function it plays in preventing confirmation of self‐critical cognitions.

**Conclusions:**

The results indicate that interventions targeting perfectionism specifically in individuals with TTM could be beneficial. Furthermore, clinicians should be sensitive to the positive functions that hairpulling can serve for people with TTM.

## INTRODUCTION

Trichotillomania (TTM) is classified as an obsessive‐compulsive and related disorder in the Diagnostic and Statistical Manual of Mental Health Disorders Fifth Edition Text Revision (DSM‐5‐TR; American Psychiatric Association, 2022). A recent systematic review of 30 studies estimated the prevalence of TTM to be 1.14% (Thomson et al., 2022). TTM is categorised as a body‐focused repetitive behaviour (BFRB) (see Najera, 2022, for a review of BFRBs). Individuals with TTM repeatedly pull out their hair, despite recurrent attempts to resist or stop pulling and the negative impact it has on their life. The repetitive nature can lead to noticeable hair loss from various sites of the body (e.g. scalp, pubic area, eyebrows) (Grant & Chamberlain, 2016). This can reduce quality of life by impacting the individual's self‐esteem and self‐perceived attractiveness (Stemberger et al., 2000; Valle & Grant, 2022), resulting in, for example, avoidance of social situations and difficulties at work (Tung et al., 2015).

Penzel (2003) proposed that hairpulling serves to externally regulate internal states of overstimulation (e.g. reducing feelings of stress) and under‐stimulation (e.g. relieving feelings of boredom). Furthermore, individuals with BFRBs are thought to have difficulty with identification and modification of negative emotions, with hairpulling being an external way to reduce these in people with TTM (Roberts et al., 2013).

Research has supported this, showing that positive and negative mood can trigger hairpulling (du Toit et al., 2001; Waites, 2020) and an altering of affect. For example, reduced feelings of frustration and anger occur through hairpulling (Siwiec & McBride, 2016), reinforcing future hairpulling episodes (Roberts et al., 2016).

The frustrated action (FA) model was developed from findings of several studies, including O'Connor et al. (2001) and Pélissier and O'Connor (2004). It proposes that perfectionistic beliefs in people who apply more effort than required (overprepare) and try to achieve too much (overactive) cause tension and frustration. These feelings develop from thoughts, such as they are not performing well enough or that they are wasting time. The model also theorises that boredom increases when individuals cannot be productive. These affective states can trigger hairpulling, decreasing those states and negatively reinforcing future hairpulling. In addition, the FA model suggests that individuals feel like they have taken action by hairpulling, positively reinforcing future hairpulling (Roberts et al., 2015).

Research has supported the FA model. In a BFRB group, Roberts et al. (2015) found boredom, frustration and impatience increased the urge to engage in BFRBs, in comparison to a non‐clinical control group. They also found higher levels of maladaptive planning (e.g. overpreparation or overactivity) in the BFRB group, which had a significant correlation with emotion regulation difficulties. However, this study included a small sample size of participants with BFRB and so interpretation of results is limited in the context of TTM specifically. The FA model suggests that it is the perfectionistic beliefs in those with high levels of maladaptive planning that cause negative affective states. Individuals find such negative affect difficult to regulate, which in turn triggers hairpulling to serve this function (Roberts et al., 2015).

In addition, existing research indicates that perfectionism plays a part in TTM (e.g. Noble et al., 2017; Pinto et al., 2017; Rehm et al., 2015). In a case study, Pélissier and O'Connor (2004) demonstrated the effectiveness of habit reversal training, with the inclusion of a cognitive component targeting perfectionistic beliefs and style of planning, in an adult female with TTM. Treatment outcomes showed a reduction in the average number of hairs pulled and feelings of tension, and an improvement in mood. This supports the notion that perfectionism has a role in TTM.

Research investigating perfectionism in people with TTM alone and not within a BFRB group is limited and, although it has been identified as having a role in hairpulling behaviour, it is not clear what that role is. The current study aimed to gain an in‐depth understanding of the role that perfectionism plays in the experiences of hairpulling in people with TTM and to explore related perfectionistic beliefs.

## METHOD

### Design

The research was conducted and reported in line with the Consolidated Criteria for Reporting Qualitative Research (COREQ; see Appendix S1 for the checklist). A qualitative semi‐structured interview was used to explore the experiences of people with TTM and their perfectionistic beliefs. The research team comprised a clinical psychologist in training (first author), two qualified clinical psychologist academics and an academic psychologist. Two members of the team also contributed to the study from a lived experience of TTM perspective and worked collaboratively with the first author from the inception to dissemination of the study.

The first author was female and had previous research experience as a research assistant and through undertaking BSc and MSc degrees. No relationships between the first author and participants were established prior to study commencement, and participants were informed of the reasons for the research in the participant information sheet.

### Participants and recruitment

Individuals eligible to participate were aged ≥18 years, self‐identified TTM to be their primary difficulty and had access to a digital device for completion of part one of the study. All participants indicated on the consent form that they did not have a diagnosis of obsessive‐compulsive disorder (OCD) or body dysmorphic disorder (BDD). Participants were excluded if they scored above the clinical cut‐offs on standardised measures of OCD (*n* = 13) and BDD (*n* = 0). Exclusion criteria also included an inability to speak English well enough to complete the study and an inability to provide informed consent. Participants were recruited via advertisements on social media and in BFRB support groups.

### Measures

To characterise the sample, participants were asked to answer a series of demographic questions (Appendix S2). Participants were also asked to complete a series of self‐report standardised measures for the purposes of screening and to further characterise the sample (Table 1).

**TABLE 1:** Standardised measures.

Standardised measure	Information	Psychometric properties
Screening measures for inclusion/exclusion criteria		
Obsessive‐Compulsive Inventory – Revised (OCI‐R) Foa et al. (2002)	Measure of OCD symptoms Higher score = greater severity Score ≥ 21 indicates OCD	Good test–retest reliability *r* = .74–.91 Good internal consistency *α* = .81–.93 Good construct validity
Body Image Questionnaire (BIQ) Veale et al. (2012)	Measure of BDD symptoms Higher score = higher levels of symptoms of BDD Score ≥ 40 indicates BDD	Good test–retest reliability *r* = .87 Excellent internal consistency *α* = .91 Good convergent validity
TTM measure		
Massachusetts General Hospital Hairpulling Scale (MGH‐HPS) Keuthen et al. (1995)	Measures of hairpulling severity Higher score = increasing severity Maximum score = 28	Excellent test–retest reliability *r* = .97 Good internal consistency *α* = .89
Affect measures		
Patient Health Questionnaire (PHQ‐9) Kroenke et al. (2001)	Measure of depression symptom severity Higher score = increasing severity Maximum score = 27	Good test re‐test reliability *r* = .84 Good internal consistency *α* = .86–.89 Good construct and criterion validity
Generalised Anxiety Disorder Scale (GAD‐7) Spitzer et al. (2006)	Measure of anxiety symptom severity Higher score = increasing severity Maximum score = 21	Good test–retest reliability *r* = .83 Excellent internal consistency *α* = .92 Good construct, criterion and factorial validity
Perfectionism measures		
Frost Multidimensional Perfectionism Scale (FMPS) Frost et al. (1990)	Measure of perfectionism Six dimensions Higher scores = higher levels of perfectionism Maximum total score = 175	Good internal consistency *α* = .77–.93 Good concurrent validity
Style of Planning Questionnaire (STOP) O'Connor et al. (2015)	Measure of maladaptive planning style in obsessive‐compulsive spectrum disorders Higher score = higher levels of maladaptive planning Maximum total score = 210	Acceptable test–retest reliability *r* = .77 Acceptable internal consistency *α* = .77 Good convergent and divergent validity

*Note*: To discriminate between TTM symptoms and BDD symptoms, potential participants scoring highly on the BIQ were presented with an additional question that aimed to identify the nature of their responses. If responses were directly related to the hairpulling, the nature of the potential participant's problem was considered to be TTM rather than BDD. These participants continued on to complete the remaining questionnaires. Potential participants were excluded if they provided a response related to another part of their body. *N* = 0 participants were excluded based on this criterion.

### Interview schedule

A semi‐structured interview schedule was developed by the first author in consultation with the research team, including those with lived experience, and was informed by relevant literature. (See Appendix S3 for the interview schedule). Drawing on lived experience within the team, the wording of questions was revised to best capture the experiences of the participants.

### Procedure

Ethical approval was granted from the University of Bath Psychology Research Ethics Committee (22‐027). The recruitment advertisement invited individuals to contact the first author via email if they were interested in participating. They were then emailed a link to a secure online portal where they could find the study's information sheet, followed by an electronic consent form. Those wishing to participate then completed two standardised measures to screen for eligibility. Participants scoring above the clinical cut‐off for OCD or BDD were excluded at this point and received a pop‐up message to explain this. It included the first author's contact details, inviting participants to make contact if they had queries or concerns. Eligible participants provided demographic information and completed the remaining standardised measures. On completion of the measures, participants were invited to select a time and date to partake in the interview. No individuals in addition to the first author and participants were present during the interviews. Interviews were conducted by the first author via Microsoft Teams, which produced a transcript of the conversation. The interviews were also audio recorded using a digital recorder, so the transcripts could be checked for accuracy. Interviews lasted approximately 45 min to 1 h in duration. On completion of the interview, participants were debriefed and provided with information signposting to further relevant support. This information was also presented to participants at the end of the online questionnaire part of the study. The option of engaging in a mindfulness exercise was also offered to participants at both time points to minimise any impact on their mood and assist with transitioning back into their day. Participants were given the option of receiving a £20 payment or e‐voucher for taking part. The first author made field notes of contextual information after each interview and initial reflections. Following review of each transcript, data was anonymised, and audio recordings were securely destroyed. Transcripts were not returned to participants for comments or corrections. No repeat interviews were carried out. Recruitment of participants after 20 interviews was stopped as sufficient information power was considered to have been obtained (Malterud et al., 2016).

### Data analysis

Descriptive statistics were used to summarise the demographic and clinical characteristics of the sample. Reflexive Thematic Analysis (RTA; Braun & Clarke, 2022) was used to analyse the transcripts. The six‐stage approach to RTA was followed iteratively. (See Appendix S4 for details on how the phases were applied.) RTA was chosen as a suitable method for answering the research question as it allows for the identification of patterns of experiences and meaning across datasets (Braun & Clarke, 2021). Field notes captured initial thoughts and reflections of the interviewer (first author), including important details about the interview and initial ideas for data analysis. These were shared in reflexive discussions with the research team and drawn upon during the analysis phase to support understanding of context. Field notes were reviewed to reflect on the first author's biases and assumptions that might have been influencing the interpretation of data. They were revisited throughout the analysis process and were considered during coding, the generation of new ideas and developing themes. The first author coded the data with the use of NVivo software version 1.7.1. A data‐driven approach to coding was employed. Semantic coding was chosen over latent coding as it reflects the explicit meanings of participant data, which is most aligned with a critical realist stance. The analysis was conducted from an ontological and epistemological stance of critical realism and contextualism. It was assumed that some reality could be found that would contribute to our understanding of perfectionism in TTM but that this discovery would be partial and influenced by what the authors brought to the research and the research context. The first author kept a reflexive diary to document how her own life experiences, knowledge, and research practice interacted with the process of analysis and interpretation of the data. For example, through the reflexive diary, she recognised her own perfectionistic beliefs and style of perfectionism, and noticed how she was drawn to data that she related to. In response, she made a conscious effort to give data that she was less drawn to the same level of detail in the analysis. In addition to the diary, the reflexivity process included a regular group supervision space where personal experiences and existing research literature were discussed. This process was engaged with across every stage of the methodology. Those of the research team with lived experience of TTM reflexively acknowledged how their personal experiences influenced their contributions to the discussions. These experiences were integrated with the reflexive contributions of the other researchers in the team. Decision making in meetings was informed by these reflexive discussions. Participants were not consulted to provide feedback on the findings. Member checking is not recommended in RTA (Braun & Clarke, 2024). RTA is a reflexive process whereby the researcher is actively part of the analysis. The analysis was conducted from a stance of critical realism and contextualism, understanding that there would be an influence of what the research team brought to the research and the research context.

## RESULTS

### Participants

Demographic and clinical characteristics of the sample are presented in Table 2. Twenty participants completed both parts of the study (i.e. the online questionnaires and the interview). Participants' ages ranged from 18 to 49 years. Two participants scored above the cut‐off on the BIQ; however, they reported that their appearance concerns were related to their TTM. Therefore, they remained eligible to continue with the study and their data was included. On average, there were only a few years between TTM age of onset (M = 11.95, SD = 5.71) and TTM being reported to significantly interfere with participants' lives (M = 15.85, SD = 6.51). On average, participants reported learning that they were experiencing TTM within a year of it significantly interfering with their lives (M = 16.30, SD = 5.60). Of the 20 participants, *N* = 17 (85%) had sought professional help for their TTM, with three participants (17.65%) reporting this to have been ‘somewhat helpful’ and *N* = 14 (82.35%) reporting that it was ‘not at all helpful’.

**TABLE 2:** Demographic and clinical characteristics of the sample.

Participant demographics	M (SD)
Age	31.05 (7.24)
	***N* (%)**
Gender	
Female	20 (100)
Ethnicity	
White British	12 (60)
White Irish	1 (5)
White Other	5 (25)
Jewish	1 (5)
Middle Eastern	1 (5)
Education	
Diploma	3 (15)
Undergraduate degree	10 (50)
Postgraduate degree	6 (30)
A‐Levels or equivalent	1 (5)
Employment	
Employed full time	14 (70)
Employed part time	2 (10)
Full time student	3 (15)
Self‐employed	1 (5)

	M (SD)	Range
Clinical characteristics		
MGH‐HPS	17.75 (4.25)	8–25
FMPS total	110.75 (15.95)	84–146
STOP total	101.05 (16.19)	71–131
PHQ‐9	7.9 (4.70)	0–19
GAD‐7	7.1 (4.31)	0–18
Screening questionnaires		
OCI‐R	10.85 (4.64)	2–20
BIQ	19.20 (11.75)	6–46

Abbreviations: BIQ, Body Image Questionnaire (Veale et al., 2012); FMPS, Frost Multidimensional Perfectionism Scale (Frost et al., 1990); GAD‐7, Generalized Anxiety Disorder Scale (Spitzer et al., 2006); MGH‐HPS, Massachusetts General Hospital Hairpulling Scale (Keuthen et al., 1995); OCI‐R, Obsessive‐Compulsive Inventory – Revised (Foa et al., 2002); PHQ‐9, Patient Health Questionnaire (Kroenke et al., 2001); STOP, Style of Planning Questionnaire (O'Connor et al., 2015).

### Reflexive thematic analysis

**FIGURE 1:**
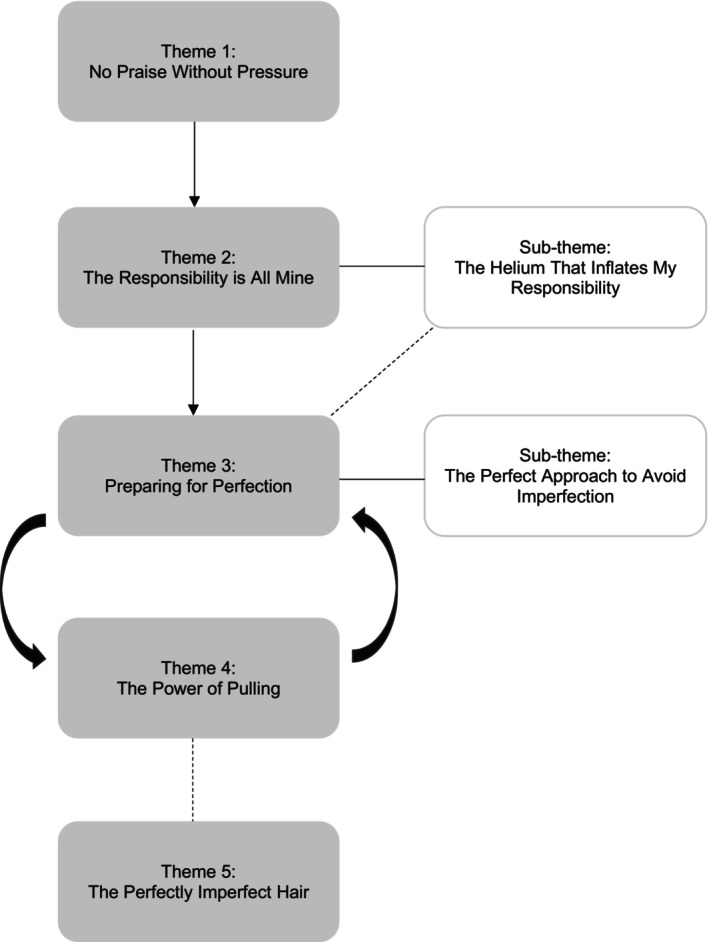
Thematic map. The thematic map is presented as a formulation. The downward arrows represent a linear development, and the curved arrows represent a maintenance cycle. The dotted lines represent a relationship between themes/sub‐themes.

Five themes and two sub‐themes were generated. The themes are presented in a thematic map in Figure 1 and include: (1) No Praise Without Pressure, (2) The Responsibility is All Mine (sub‐theme: The Helium That Inflates My Responsibility), (3) Preparing for Perfection (sub‐theme: The Perfect Approach to Avoid Imperfection), (4) The Power of Pulling and 5) The Perfectly Imperfect Hair.

#### Theme 1: No praise without pressure

Across the dataset, memories of childhood experiences were commonly of being a child and feeling the weight of expectations from adults and authoritative systems (i.e., parents, church, and teachers). Participants described moral expectations pertaining to rigid beliefs about what was right and wrong, expectations to follow rules and behave well, and academic expectations to perform well at school. If these expectations were met, they would receive approval. However, the bar for meeting such expectations was not only high but for some was potentially unattainable without continuous striving.

I grew up in a very toxic church, as well, that had very strong views on things, like you know, my sexuality, […] it was this whole environment of like if you go out with a non‐Christian person, you're no longer allowed any sort of leadership role within the church. (Annie)

There's a certain culture and element to ballet that's very strict on discipline and perfectionism […] You constantly have to think about refining your technique and like self‐talk about being better and better and better and you're never kind of good enough […] and then when you were in class you had to be very quiet and follow a lot of rules. (Mia)

… if I did something wrong, what I remember is they [parents] would be like “oh we expected better from you” […] we had a good relationship, but I always felt like there was this expectation of me of being, yeah, perfect, like, not … being allowed to make mistakes even though my brother was allowed to…. (Kim)

Many participants not only described feeling pressure from others but also the process of internalising the pressure to live up to others' expectations. At times when participants felt they had not met or exceeded the expectations of others, a sense of disappointment in themselves for not doing better was described. The responsibility beliefs that participants developed by internalising the pressure from others forced them to step into an adult role, while still only a child.

I really picked up on the dynamics at home, and my mum, in a certain sense it feels, almost evangelised me a bit like as this wonderful child […] I think drove me to be good and be that like “oh you're so mature for your age, you're such a wonderful little girl” and you know all of that sort of stuff, because I didn't want to add to the distress that was already there at home. (Rosie)

I used to go, “right, okay, my mum and my dad think I've got the… she's [mum] got this particular expectation of me, she wants me to do really well, she wants me to go to university, she wants me to do the best I can and get those A grades” and then when I wouldn't, I beat myself up over it. (Evelyn)

#### Theme 2: The responsibility is all mine

The internalising of the pressure and expectations perceived to be prescribed to the individual by significant others (theme 1) in turn led to an inflation of personal responsibility as an adult. Across the data, there was a belief that the participants were personally responsible for situations that were objectively outside of their control. Specific to TTM behaviour, most participants reported that they were aware of how their hairpulling persisted despite their repeated attempts to stop. This knowledge was underpinned by a sense of feeling responsible for the behaviour. As such, an episode of hairpulling was followed by feelings of shame, regret and guilt accompanied by self‐blaming self‐talk.

I lost complete control and I just pulled and pulled and pulled and I ended up quite badly clearing my upper lash line on both eyes and there was like a very brief moment of satisfaction and then there was again that regret of “I've done this to myself” like, “why am I doing this to myself, why am I putting myself through this?” (Isabelle)

For some participants, their feelings of shame and responsibility for hairpulling prohibited them from sharing with others that they had TTM. They did not want others to know that they had pulled their own hair out.

I would never say “oh I have trich” or “I pulled it out”, it's like “oh I'm just going under a lot of stress and I'm dealing with hair loss” not like that I'm taking an active role in it, because it felt bad to like, tell people that, like, “yeah, this is my fault, I'm the one who's doing this” (Sophia)

A sense of inflated responsibility was also evident in situations involving other people. Many participants talked about wanting to please others and to not let others down. For example, Jade reported ‘I just want to please and in life you can't please everyone… and logically that is something that I know but… something in me is like you know “if you really try you might be able to”’. Despite rationally knowing that they could not please everyone, there was an internal sense that it was still possible to do so and a belief that they might possess the qualities to allow them to do this. The internalised pressure from living up to others' expectations as children influenced participants' beliefs that if they tried hard enough, they could please others. When it was self‐perceived that their own actions had let others down, it was interpreted as a personal failing. Furthermore, for some participants this inflated sense of responsibility underpinned the belief that uncontrollable situations could, in fact, have been controlled, if only they had made better decisions. Therefore, when the uncontrollable happened, this was again interpreted as a personal failing. Letting others down could have been avoided or prevented if they had acted differently.

It could literally be something like I've organised a picnic, I've put all my heart and soul into it, and it has rained and that would be my fault because I chose to do a picnic outside. If I had chosen to do it inside…. (Annie)

#### Sub‐theme: The helium that inflates my responsibility

This sub‐theme falls under theme 2 and highlights what can be identified as the power source for the individual's responsibility beliefs and interpretations. This is a pattern of negative self‐beliefs and self‐critical talk, as reported by almost all participants. Many participants tied their self‐worth to doing well in tasks and felt like a failure if something did not go according to plan.

If I made a mistake, I would punish myself mentally by saying “oh I am such a failure, I'm worthless, I'm never gonna do anything with my life, no one wants me, no one likes me” like I would just mentally screw off myself because I made a mistake. (Evelyn)

By making mistakes, participants' negative self‐beliefs are confirmed and exposed to others, as described by Anita, ‘I'm gonna cry now… that I'm a bit hopeless, I'm worthless and I failed…yeah…you know, embarrassment as well because I worry that people think I'm stupid’. Participants talked about the catastrophic consequences of making mistakes. For example, Zoe shared, ‘I'll just convince myself that I'm gonna lose my job and I'm not gonna be able to provide for my family and it's just gonna be really bad’. The perceived gravity of not doing something to the best of their ability at all times and in all situations is reflected in the resulting negative self‐evaluations and perceived catastrophic consequences.

#### Theme 3: Preparing for perfection

To prevent making mistakes that would confirm negative self‐beliefs, participants engaged with the planning and organising of tasks by either approach or avoidance. Both are conceptualised here as safety‐seeking behaviours, as the goal is to prevent making mistakes and, in turn, avoid confirming their self‐critical cognitions. Whether the behaviour was to plan ahead or leave tasks to the last minute, tasks needed to be done well. Both strategies lead to an increased urge to hairpull.

#### Sub‐theme: The perfect approach to avoid imperfection

Approach and avoidance of tasks could be viewed as the anchors at either end of a spectrum, both with the same function of avoiding confirmation of self‐critical cognitions. When approaching tasks, some participants adopted the strategy of being organised, planning ahead and carrying out their work thoroughly and with precision, as described by Emma, ‘I hate making mistakes, I am a bit of a perfectionist, I'll spend a long time doing things, I don't rush things generally’. This strategy was associated with an intolerance of uncertainty. Participants described needing to overprepare to deal with potential unknown situations and in case something went wrong. Planning ahead provided a sense of control in enabling situations to go well and, ultimately, avoiding confirmation of their self‐critical cognitions.

…I'll want to forensically go over something and make sure I'm as prepared as possible because I'll worry about, “what if a patient asks me something that I don't know the answer for?” Or it's dealing with the unknown, I'm not a big fan of dealing with unknown things. (Helen)

If I were to host a get together with my friends, I am that person who will go way over the top […] maybe we'll like, go see a movie, but if the movie turns out to not be good, then we can go back to my house and play a game and here are some different options we have, things like that so that if it doesn't work out well there's like a backup, like a backup for the backup, I definitely overprepare for social events like that just ‘cause I'm worried it'll go wrong’. (Sophia)

When avoiding tasks, many participants described leaving tasks to the last minute and procrastinating, despite the stress that would accompany this strategy. The fear of making a mistake and producing something not worthy prevented them from starting tasks. Despite leaving tasks until there was little time to complete them, participants still described the desire to complete the tasks well.

I procrastinate on stuff and then it just ends up in a world of like stress and then hair pulling and it's not good. Like if I'm writing an essay for a class […] and I'm doing it 2 h before it's due at midnight. (Siobhan)

I wasn't being productive, I was becoming counterproductive and not getting things done, which is then putting added stress on me because I wasn't meeting my deadlines, but it's because I didn't want to make a mistake and I did want it to be good. (Emma)

#### Theme 4: The power of pulling

This theme captures a pattern in the data that highlights the positive functions that hair pulling can serve. Hairpulling functioned to support the individual to cope with, or navigate, the feelings of anxiety or frustration when they were engaging in the safety‐seeking behaviours as described above in *The Perfect Approach to Avoid Imperfection*. When the strategy was to approach tasks ahead of time, hairpulling helped the individual to focus their attention on an important task for a sustained period.

I always thought it helped me cope with focus, so when I was sitting in my room working on homework for a couple hours and like I would lose my focus and my stamina and I would resort to just like fidgeting with my skin or my eyelashes or my eyebrows. (Lucy)

I was focusing on a task or […] doing some analytical work […] when I'm focusing hard and sometimes I'm really enjoying the task at hand, but I definitely want to make sure I do it right, it's often when I care a lot about it and I'm focused. (Mia)

For those delaying or avoiding tasks hairpulling provided a crutch, acting as a coping mechanism, serving to calm them when feeling anxious. Pulling was also a form of procrastination that supported individuals by making them feel busy when they were not actively carrying out a task that they believed they should have been.

When I feel anxious, I will pull more, so in those situations [leaving tasks to the last minute] I will be nervous and I will be anxious… sometimes I know I'm doing it, and sometimes I don't and I have noticed that my husband would tell me like “you're doing it” and I've not even realised and that's when I am quite anxious and stuff and I almost feel annoyed that he's telling me to stop because I don't want to because when I feel like that it helps me cope. (Grace)

I think it's often when I'm relaxing and I shouldn't be relaxing like I should be doing something else…and it's almost like a… it provides a function to be busy without being busy […] I used to think that I used to stress pull but I don't think I'm pulling because I'm stressed, I think I'm stressed that I'm not doing what I think I should be doing. (Annie)

By engaging with the urge to pull, the safety‐seeking behaviour of approach or avoidance is supported to continue, and confirmation of self‐critical cognitions is avoided. The positive function of hairpulling reinforces future episodes of hairpulling. Alternatively, if the individual does not pull, they would be faced with the risk of increased anxiety/difficulty focusing. In their view, this would likely contribute to the probability of making mistakes and a decrease in their ability to produce something at their best, confirming to the individual that they are a failure and the worst is going to happen.

#### Theme 5: The perfectly imperfect hair

This theme encapsulates a pattern across the dataset whereby participants described imperfect hairs as the ‘perfect’ hairs to pull. These hairs were the ones that felt out of place or felt like they did not belong, for example, they might have a different texture to their other hairs, or they might have a bigger root. These hairs were not how the individuals felt they should be and so they were the hairs targeted to pull.

They tend to be coarser thicker hairs for me… and when I've spoken with other people who have trich, they also tend to say this, it is like there's a different texture, it feels a bit different, it's almost like it's out of place, like it doesn't belong with the rest of your hair. (Kim)

Sometimes the perfect hairs to pull were searched for in an attempt to achieve a specific feeling. This feeling was conceptualised by participants as a ‘perfect feeling’ (e.g. Laura) or just right feeling.

It's about finding the right hair… thing is that until you get the chance to speak to someone else about it, that understands what it is you're going through, you don't realise that actually that is what it is… it is that you're looking for that one that feels just right. (Isabelle)

Participants reported that although there was a feeling of satisfaction associated with pulling the perfect hairs, this feeling lasted ‘not very long, so probably seconds, if not minutes at the most, and then you either feel guilty or you just move on to the next hair’ (Grace). Hannah further explained that ‘by the time I get that hair that I think is just right I'm really just very much in the cycle of pulling’, suggesting that the function of hairpulling changes during an episode and perhaps alters from focused pulling to pulling that is less in the individual's conscious awareness.

## DISCUSSION

This study aimed to gain an in‐depth understanding of the role that perfectionism plays in the experiences of hairpulling in people with TTM and to identify their perfectionistic beliefs. A formulation of the themes was developed, highlighting a continuous role of perfectionism intertwined throughout the process of TTM, from childhood through to the present experience of hairpulling. Within this formulation, a maintenance cycle is proposed, explaining how hairpulling supports the individual in engaging in behaviours that prevent outcomes confirming negative self‐beliefs. In addition, mechanisms of TTM were evident in the data that have also been found to play central roles in other disorders (Coles et al., 2003; Dugas et al., 2022; Mofrad et al., 2020; Salkovskis, 1999).

Results of the current study add to the existing literature by explaining a specific process of maladaptive perfectionism relevant to experiences of TTM and how it develops. Shafran et al. (2016) outlined that maladaptive perfectionism involves self‐critical evaluation and extreme concern about making mistakes, leading to self‐defined rules and actions which function to avoid the perception of failure. The current study recognises this process in people with TTM as developing from early childhood experiences of pressure to live up to others' expectations to gain their approval (*No Praise Without Pressure*). The inflated sense of responsibility developed from these experiences (*The Responsibility is All Mine*) and was underpinned by self‐critical cognitions (*The Helium That Inflates My Responsibility*). The proposed maintenance cycle describes the actions (*Preparing for Perfection*; *The Power of Pulling*) functioning to avoid the perception of failure.

In association, the maintenance cycle can be partially explained by the CBT Model of Low Self‐Esteem (Fennell, 1997). According to this model, core beliefs about the self, others and the world are developed through the individual's life experiences (*No Praise Without Pressure*). With low self‐esteem, core beliefs about the self are negative, and described as the ‘bottom line’ (Fennell, 1997). The bottom line in the current data (*The Helium That Inflates My Responsibility*) appears to often be ‘I am worthless’ or ‘I am a failure’. To protect themselves, individuals live by certain standards (not making mistakes, not letting others down) and when the individual perceives these standards are not or will not be achieved, the bottom line is confirmed. To avoid confirmation of the bottom line, participants either overprepared and planned ahead to achieve their standards or delayed starting tasks and procrastinated so that they did not have to face the potential for not achieving their standards (*The Perfect Approach to Avoid Imperfection*). Several studies have shown a relationship between low self‐esteem and TTM, and significantly higher levels of low self‐esteem in people with TTM than without TTM (Diefenbach et al., 2005; Soriano et al., 1996; Stemberger et al., 2000), supporting the use of this model as a credible explanation for underlying processes.

In the current data, perfection was also evident in the specific act of hairpulling, engrained in the experience and type of hair pulled for satisfaction from pulling to be achieved (*The Perfectly Imperfect Hair*). This finding overlaps with the results of a previous qualitative study investigating hairpulling rituals in people with TTM (Waites, 2020). Waites found that participants did not pull hair randomly; instead, pulling was organised by beliefs and learned information about how to enhance the regulating function of TTM.

The findings of this study only partially support the FA model. The results show that perfectionistic beliefs are involved in the maintenance of hairpulling behaviour. However, the RTA showed that not all participants adopted an overactive and overprepared style of planning, with some avoiding starting tasks until they were almost out of time to complete the task. In support of the model, pulling served to support the individuals with their safety‐seeking behaviour, reducing feelings of tension and frustration. However, although taking action by pulling did positively reinforce future episodes of pulling, it is likely that the positive outcome of sustained attention/calming anxiety was the reinforcer, rather than the feeling of taking action itself.

Interesting to note from the current analysis is the presence of mechanisms that have been suggested to play central roles in other disorders. For example, in OCD, inflated responsibility is suggested to be a key mechanism, in which the individual believes their actions (or lack of) will adversely impact themselves or others and that they can prevent this (Salkovskis, 1999). In addition, Coles et al. (2003) suggested that individuals with OCD carry out rituals to reduce the discomfort of imperfect sensations, known as Not Just Right Experiences (NJREs). Furthermore, intolerance of uncertainty has been identified as a key mechanism of Generalised Anxiety Disorder (GAD). In a RCT investigating a treatment specifically targeting intolerance of uncertainty in GAD, Dugas et al. (2022) demonstrated significant improvements compared to a waitlist control group. Moreover, a recent study demonstrated intolerance of uncertainty as a transdiagnostic concept, underpinning the maintenance of many psychological disorders (Mofrad et al., 2020). In relation to the current data, participants reported that they felt responsible for situations outside of their control, particularly when others were impacted (inflated responsibility), needed to pull the hairs that felt like they did not belong (NJREs), and would prepare for all possible scenarios to avoid not knowing what to do if something went wrong (intolerance of uncertainty). Findings suggest that these are all transdiagnostic mechanisms as opposed to disorder specific.

### Strengths and limitations

Whilst perfectionism has previously been associated with TTM (e.g. Grant et al., 2021; Noble et al., 2017; Pinto et al., 2017), this is the first qualitative study to investigate the experience of perfectionism and its role in TTM. Conducting interviews allowed for gathering rich data, with which to conduct an in‐depth analysis of participant experiences and perfectionistic beliefs. Furthermore, this study is the first to highlight how perfectionism is intertwined throughout the process of TTM, from childhood. In addition, it proposes a maintenance cycle of hairpulling, within the context of perfectionism, with potential clinical utility.

A key strength of this study was the lived experience expertise of TTM within the research team. The incorporation of perspectives from a lived experience position was included in the decision‐making process from the study's inception through to the write‐up. Such discussions throughout the research process enabled the first author to keep it as relevant as possible to people with TTM and gain a better understanding of how the findings related to real‐life experiences of those experiencing TTM. Furthermore, the final themes resonated with the members of the research team who had lived experience of TTM, reflecting a degree of credibility of the findings and giving the reader confidence in the interpretation of results.

The strengths of the current study should be interpreted with the following limitations in mind. Participants were included if they perceived TTM to be their most significant problem but were not required to have a diagnosis. Therefore, it is possible that the sample included chronic hairpullers not meeting full diagnostic criteria for TTM. However, TTM continues to be an under‐recognised condition, with many individuals meeting DSM‐5‐TR diagnostic criteria (American Psychiatric Association, 2022) who have not sought treatment or whose condition has not been identified by professionals. Therefore, the research team and first author decided that a subjective confirmation of TTM would be appropriate.

Participants in this study were mainly recruited via social media, allowing opportunity for a diverse range of people from different cultures and communities to take part. However, all participants in this sample were female and mostly white. Despite this being common in existing TTM research, recent studies have found that TTM is not more common in this gender or ethnic group (Grant et al., 2020; Thomson et al., 2022). The absence of male participants may have impacted the role of perfectionism described in this study. There are different societal norms regarding hair loss between males and females, and there may be differences in expectations placed on themselves and by others. Therefore, it is possible that there may also be a difference in the way that TTM is experienced. Furthermore, the current sample may reflect a population more likely to participate in studies and have access to digital devices. It is possible that those with low socio‐economic status or fewer educational years might not participate. Future research should aim to investigate TTM in a more diverse and representative sample of the TTM population and consider barriers to participation.

During the interviews, two participants reported having a diagnosis of attention deficit hyperactivity disorder (ADHD). Recent studies in the USA have found varied prevalence rates of TTM with comorbid ADHD: 15.3% (Chesivoir et al., 2022) and 29% (Grant et al., 2020), both much higher than the prevalence of ADHD in the general population (6.76%; Song et al., 2021). On reflection, it would have been useful to screen for ADHD in the current study. It is possible that the way individuals with ADHD approached tasks was impacted by ADHD characteristics, such as poor planning and time management skills, and not only linked to facets of perfectionism, such as a fear of making mistakes. This may have been misinterpreted in the data as procrastination and avoidance. Future research might screen for ADHD, but with a high prevalence in the TTM community, it would be advantageous to compare results between those with and without ADHD, rather than excluding this group.

Finally, this was a qualitative study with the aim of gaining an in‐depth understanding of the role that perfectionism plays in the experiences of hairpulling in people with TTM, and to identify their perfectionistic beliefs. While the study sheds some light on this phenomenon from the subjective experiences of individuals with TTM, it is important to acknowledge that there are likely to be individual differences in the psychological mechanisms that underlie TTM.

### Clinical implications

To date, there is minimal research on the use of interventions targeting perfectionism specifically in individuals with TTM. The current study indicates that these could be useful interventions. It did not require participants to have perfectionism, and some participants did not explicitly express having perfectionistic traits; however, traits were found in all participants, suggesting that it is a key mechanism. Furthermore, in the proposed maintenance cycle, the safety‐seeking behaviours adopted to avoid confirming self‐critical cognitions associated with perfectionistic thinking increase the risk of hairpulling urges, and so intervening by targeting perfectionism may have promising results. Additional research is required to investigate this further.

An important aspect of this study is the finding that there is some power in hairpulling and it has been perceived by some individuals with TTM to have an adaptive function. Those without TTM tend only to see the destruction caused by hairpulling and are less aware of the positive functions that it serves for some individuals. It is important for clinicians to not solely focus on the distress that hairpulling can cause the individual and to explore if there are also perceived helpful aspects. In addition, the individuals with lived experience of TTM in the research team described TTM as part of their identity, as it provided a means to support functioning. Clinicians should be sensitive to this possibility when exploring the impact of TTM on the individual.

## CONCLUSION

The findings highlight the involvement of maladaptive perfectionism in hairpulling behaviour and how it develops from childhood. A maintenance cycle is proposed in which pulling provides a positive function for avoiding confirmation of self‐critical cognitions. These have clinical implications for the treatment of TTM in people presenting with perfectionistic traits, suggesting that targeting perfectionism and formulating the way in which people with TTM approach tasks may be beneficial. There is limited research in this area, so further investigations would be useful for testing this in clinical practice.

## AUTHOR CONTRIBUTIONS

**Amy L. Zarandi:** Conceptualization; methodology; formal analysis; investigation; data curation; visualization; project administration; writing – original draft; writing – review and editing. **Josie F. A. Millar:** Conceptualization; methodology; formal analysis; writing – review and editing; supervision. **Erin Waites:** Conceptualization; methodology; formal analysis; supervision; writing – review and editing. **Judith L. Stevenson:** Conceptualization; methodology; formal analysis; supervision; writing – review and editing.

## CONFLICT OF INTEREST STATEMENT

The authors declare no conflict of interest.

## Supporting information


Appendix S1

Appendix S2

Appendix S3

Appendix S4

## Data Availability

The data that support the findings of this study are stored in the University of Bath Research Data Archive. The data are not publicly available due to privacy or ethical restrictions and can be requested by bona fide researchers via https://doi.org/10.15125/BATH‐01329 (the link will be activated once the article is published).
