# Supplementary figures and images for: A qualitative study exploring the role of perfectionism in trichotillomania
Source: Psychol Psychother. 2025 May 16;98(4):901–17. doi: 10.1111/papt.12597 (PMC12617469; doi:10.1111/papt.12597)

**Appendix S1**

**
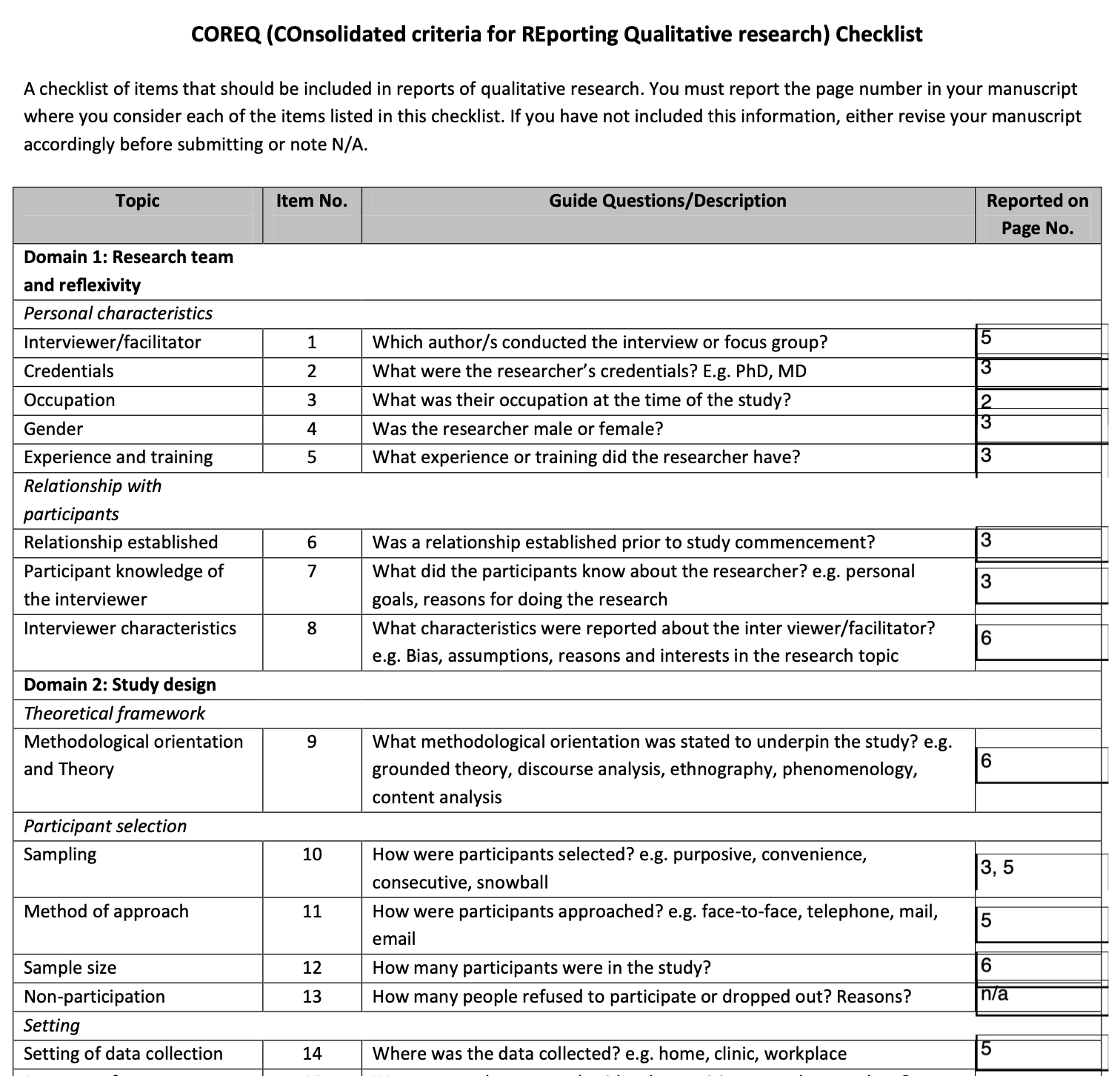
**

**
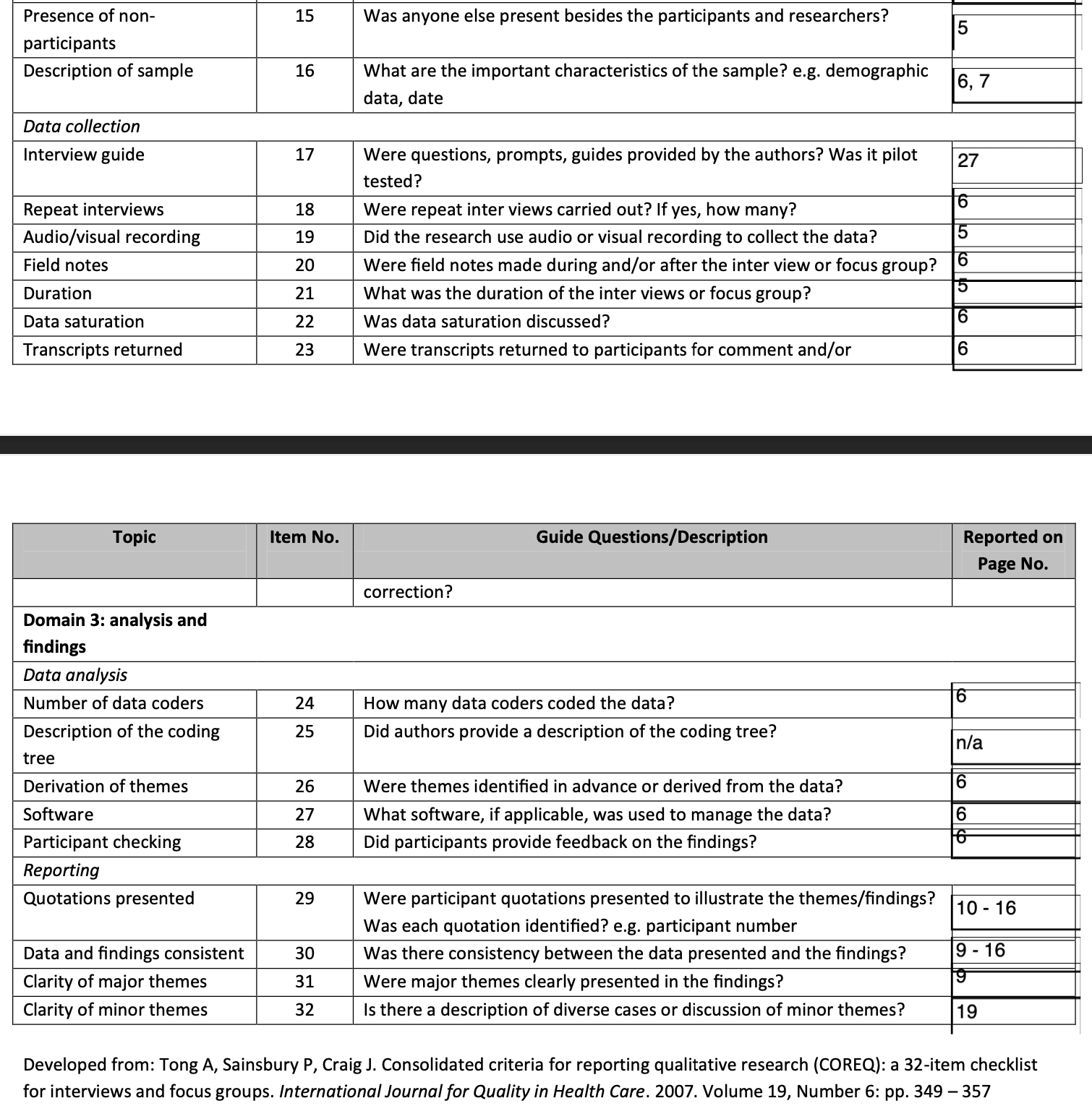
**

Supplement: Supplementary file 1 — Appendix S1 [file PAPT-98-901-s001.docx]
