# Supplementary material for: A qualitative study exploring the role of perfectionism in trichotillomania
Source: Psychol Psychother. 2025 May 16;98(4):901–17. doi: 10.1111/papt.12597 (PMC12617469; doi:10.1111/papt.12597)
Supplement: Supplementary file 2 — Appendix S2 [file PAPT-98-901-s004.docx]

**Appendix S2**

**Demographic Questions**

*The following questions will be inputted into Qualtrics. Participants will be required to tick the relevant answers to indicate their response and there will be space to type their response if a qualitative answer is required.*

**Please answer the following questions about yourself. We are asking these to gain an understanding of the overall characteristics of the study participants.**

1. What is your age?

2. Which gender do you identify with most?

Female

Male

Non binary

Other (please describe)

Prefer not to say

3. What is your ethnic group?

Bangladeshi

Indian

Pakistani

Other Asian

Black African

Black Caribbean

Other Black

Chinese

Mixed – White and Asian

Mixed – White and Black African

Mixed – White and Black Caribbean

Other Mixed

White British

White Irish

Other White

Any other (please specify)

4. What is the highest level of qualification that you have?

No qualifications

CSEs or equivalent

GCSEs or equivalent

A levels or equivalent

Diploma

Undergraduate degree

Postgraduate degree

Other (please specify)

5. What is your current employment status?

Unemployed

Not able to work

Employed – Full time

Employed – Part time

Self-employed

Full time student

Part time student

Retired

Other (please describe, e.g., homemaker, carer)

6. How old were you (approximately) when your trichotillomania began?

7. How old were you (approximately) when you first found out your problem was trichotillomania?

8. How old were you (approximately) when your trichotillomania started to significantly interfere with your life?

9. Have you ever asked for help for your trichotillomania from your GP or other health professional/mental health service?

Yes

No

10. If yes, how helpful was the treatment you received in helping you tackle your trichotillomania?

Not at all helpful

Somewhat helpful

Not at all helpful

10. How severe do you perceive your trichotillomania to be?

*Participants will be asked to indicate this on a 0-100 scale with 100 being the most severe.*
