# Supplementary material for: A qualitative study exploring the role of perfectionism in trichotillomania
Source: Psychol Psychother. 2025 May 16;98(4):901–17. doi: 10.1111/papt.12597 (PMC12617469; doi:10.1111/papt.12597)
Supplement: Supplementary file 3 — Appendix S3 [file PAPT-98-901-s002.docx]

**Appendix S3**

**Semi-Structured Interview Schedule**

Thank you for speaking with me today. Before we start, I just want to check a few things with you. You have previously participated in part one of this study by completing an online questionnaire. This interview today is part two of the study.

Before you participated in part one of this study, you provided consent to take part via a consent form. Can I please confirm with you if you still consent to take part in this interview?

Thank you. The consent form also stated that Microsoft Teams will produce a document with a transcription of your responses to the interview questions. Is this still ok?

Thank you. It also stated that the interview would be audio recorded so that the transcription could be checked for any errors and then analysed anonymously. The consent form that you completed in part one of this study indicates that you consent to the interview being recorded. May I please confirm if you still consent to have this interview recorded today?

Thank you. Once I have started the recording, I will ask you those questions again so that I have a record of your consent today.

Do you have any queries or questions for me before we begin the interview? Feel free to ask me any questions you have at any point during the interview. If you want to stop or take a break at any time that is absolutely fine, just let me know. You have the right to stop this interview and withdraw from the study at any point. If at any point during the interview, you start to feel upset or distressed by the content of what we are talking about, do let me know and, again, we can take a break or stop the interview completely.

[If joined with video] Would you like us to keep our cameras on for the interview or would you feel more comfortable turning them off? You can change your mind at any point during the interview.

I will start recording now and turn on transcribing.

Could you please confirm that you give consent to Microsoft Teams transcribing your responses to the questions?

Could you please confirm that you give consent for this interview to be audio recorded?

Thankyou. Are you ready to begin?

1. I can see from your questionnaire you started pulling at [AGE], could you tell me a bit about what was happening for you then?

2. What did it mean to you and your family when you first started to pull out your hair?

Prompts:

a. How did others respond?

b. Were there any cultural or religious influences?

c. Has this meaning changed over time?

3. Sometimes people with trichotillomania have talked about needing to pull hair until it is ‘just right’. For example, pulling until they pull a hair that has a thicker root, or seeing a hair out of place and needing to remove it. Do you ever get that just right feeling?

Prompts:

a. How long does it last?

b. What feelings come up for you while you are trying to chase that feeling of pulling a hair that is just right?

4. Tell me about how you prepare for tasks, for example, in work, in school, if hosting etc.

Prompts:

a. What kind of tasks would you be more likely to overprepare for?

b. What would it mean for you to make a mistake?

c. What feelings does it bring up if you make a mistake?

5. Think about your average day. How does having trichotillomania affect you in your everyday life?

Prompts:

a. How do you feel your hair/eyebrows/eyelashes need to look in order to do the activities you want to do?

b. What would it mean if you couldn’t get your hair/eyebrows/eyelashes to look like that?

c. Tell me about any activities you avoid doing due to hairpulling.

Thank you so much. Before we finish, I wondered if there was anything else you wanted to add that we have not spoken about?

How have you found the interview today? Do you have any questions?

I would like to remind you of the services we mentioned on the debrief form when you participated in the online questionnaire. Would you like me to share this information again with you now, or send it to you?

When you participated in the online questionnaire, there was an opportunity to take part in an exercise to help people transition out of the study and manage any difficult feelings it may have brought up. I am wondering if you would like me to guide you through a 5-minute exercise to signify this study coming to an end?

As a thank you for taking part in this project, I would like to offer you a £20 e-voucher or PayPal payment. You provided an email address in the first part of the study, can you confirm if this is the email address to send receipt of voucher/payment or if you would like to provide an alternative email address?

Thank you for taking part in this project. Your contribution is very much appreciated.
