# Supplementary material for: A qualitative study exploring the role of perfectionism in trichotillomania
Source: Psychol Psychother. 2025 May 16;98(4):901–17. doi: 10.1111/papt.12597 (PMC12617469; doi:10.1111/papt.12597)
Supplement: Supplementary file 4 — Appendix S4 [file PAPT-98-901-s003.docx]

**Appendix S4**

**The Six Phases of RTA**

| Phase | Process of Analysis |
| --- | --- |
| 1. Familiarisation with the dataset | The first author listened to each interview recording once and read each transcript twice. After each read, notes of initial thoughts and ideas were made for each data item across the dataset. |
| 2. Coding | Coding the data was facilitated with the use of NVivo software version 1.7.1. An inductive (data-driven) approach to coding was employed. Each transcript was coded twice. Transcripts were coded in a different order each time to promote a balance in depth of coding. |
| 3. Generating initial themes | Codes with shared meanings were grouped together to form candidate themes. This was achieved by creating thematic maps to understand patterns and connections. The first author re-grouped codes and re-drew thematic maps several times to explore patterns. These were shared and reflected on with the research team. |
| 4. Developing and reviewing themes | The candidate themes were checked against the coded data extracts and the full dataset to make sure that they were sufficiently supported by the data. In consultation with the research team, some revisions of candidate themes were made to redefine the boundaries of these themes. |
| 5. Refining, defining and naming themes | A thematic map was developed to understand the connections between the themes and the story they told to answer the research question. The first author wrote brief definitions of each theme to clarify the central organising concepts. The map, definitions and theme names were reviewed in consultation with the research team. |
| 6. Writing up | As the first author wrote up each theme, further refinements were made, and previous phases were revisited to determine whether the data was being sufficiently represented. |

*Note:* These six phases were not carried out in a linear fashion and involved a recursive process.
